# A pediatric patient with chronic enteropathy associated with SLCO2A1 who underwent multimodal treatment including several surgeries: a case report

**DOI:** 10.3389/fsurg.2025.1516960

**Published:** 2025-06-19

**Authors:** Yoojin Jung, Jaehee Chung, Inhyuk Yoo

**Affiliations:** ^1^ Division of Pediatric Surgery, Department of Surgery, Seoul St. Mary’s Hospital, College of Medicine, The Catholic University of Korea, Seoul, Republic of Korea; ^2^ Department of Pediatrics, College of Medicine, The Catholic University of Korea, Seoul, Republic of Korea

**Keywords:** protein-losing enteropathies, ulcer, intestine, small, capsule endoscopy

## Abstract

**Introduction:**

Chronic enteropathy associated with SLCO2A1 gene (CEAS) is a rare protein-losing enteropathy primarily recognized in Asia. Its uncommon nature and limited research usually complicate diagnosis and treatment. This review examines the course of a pediatric patient with CEAS, who underwent three surgeries during medical treatment.

**Case presentation:**

A 12-year-old girl was referred for significant anemia and hypoalbuminemia during evaluation for short stature. Initial lab results included hemoglobin of 6.1 g/dl, normal CRP, and positive stool tests, without hematochezia. Capsule endoscopy revealed chronic ulcers and strictures in small bowel, and genetic testing identified a variant in SLCO2A1 gene, finally confirming CEAS. Because the capsule kept retained for 19 days, surgical removal was performed. Alongside the incision made at ileum, extensive circular stenoses were observed. Postoperatively, the patient was started on steroid and Azathioprine. After three months, she visited the emergency room with abdominal pain and fever. CT revealed diffuse free air and abscess, but no definite perforation was identified during emergency surgery, suggesting it was sealed-off. Two weeks after discharge, infliximab treatment was initiated. But she returned with vomiting a few days after second infusion. CT showed small bowel ischemia due to closed-loop obstruction, prompting urgent surgery. Multiple fibrotic bands were twisting part of jejunum, but the strictures seemed nearly normalized compared to earlier findings. We concluded that her disease was not worsening, and the last surgery was rather due to postoperative adhesions.

**Discussion:**

This case highlights the challenges in early diagnosis of CEAS, given its rarity and nonspecific symptoms. However, it should be included in differential diagnosis for atypical clinical findings, with genetic testing as a potential diagnostic tool. Also, long-term immunosuppressive therapy often leads to complications requiring multiple surgeries, so minimally invasive approaches should always be considered. Additionally, the resolution of circular stenosis seen in the final surgery during infliximab treatment indicates a reversible component. Further research for effective treatment for CEAS is essential.

## Introduction

Chronic enteropathy associated with SLCO2A1 gene (CEAS) is a rare type of protein-losing enteropathy that has recently been recognized primarily in Asian patients particularly from Japan. Since CEAS is not a common disease and further research is needed on the differential points related to clinical manifestations, imaging, and pathological findings, it remains a challenging diagnosis for clinicians. Additionally, there is still no established consensus on appropriate treatment. It takes long durations before patients achieve even minimal therapeutic benefits, thereby multiple times of surgery is often necessary. In this case report, we aim to review the overall course of a 12-year-old female patient who, after being diagnosed with CEAS, required surgical intervention three times while receiving medical treatment.

## Case presentation

A 12-year-old female child was referred to our hospital for nutritional deficiency evaluation. Due to short stature, she had visited a local clinic right before, where tests revealed severe Iron deficiency anemia (IDA) and hypoalbuminemia. Her height was 121.5 cm, which was below the 3th percentile (3th percentile; 138.1 cm). Her father's height was 176 cm, in the 75th percentile; mother's height was 157 cm, in the 25th percentile; older brother's height was 170 cm, in the 50th percentile. She had no notable history, except for the ibuprofen treatment she received for PDA after birth. Upon taking medical history interview, she reported experiencing intermittent abdominal pain once or twice every few months for the past four years, but there were no instances of diarrhea or hematochezia. However, the patient had not received any treatment for these long-term symptoms. Initial blood tests revealed Hb 6.1 g/dl, Albumin 2.8 g/dl, Total protein 4.5 g/dl, Iron 9 mcg/dl, Ferritin 3.3 ng/ml, and TIBC 288 mcg/dl. CRP and ESR level were normal, and fecal tests showed positive results with fecal calprotectin at 78 μg/g and occult blood at 3,769 ng/ml. The initial upper endoscopy and colonoscopy showed no significant findings, but stool occult blood continued to be positive, and the IDA was refractory to iron supplements, prompting further evaluation for gastrointestinal causes.

**Figure 1:**
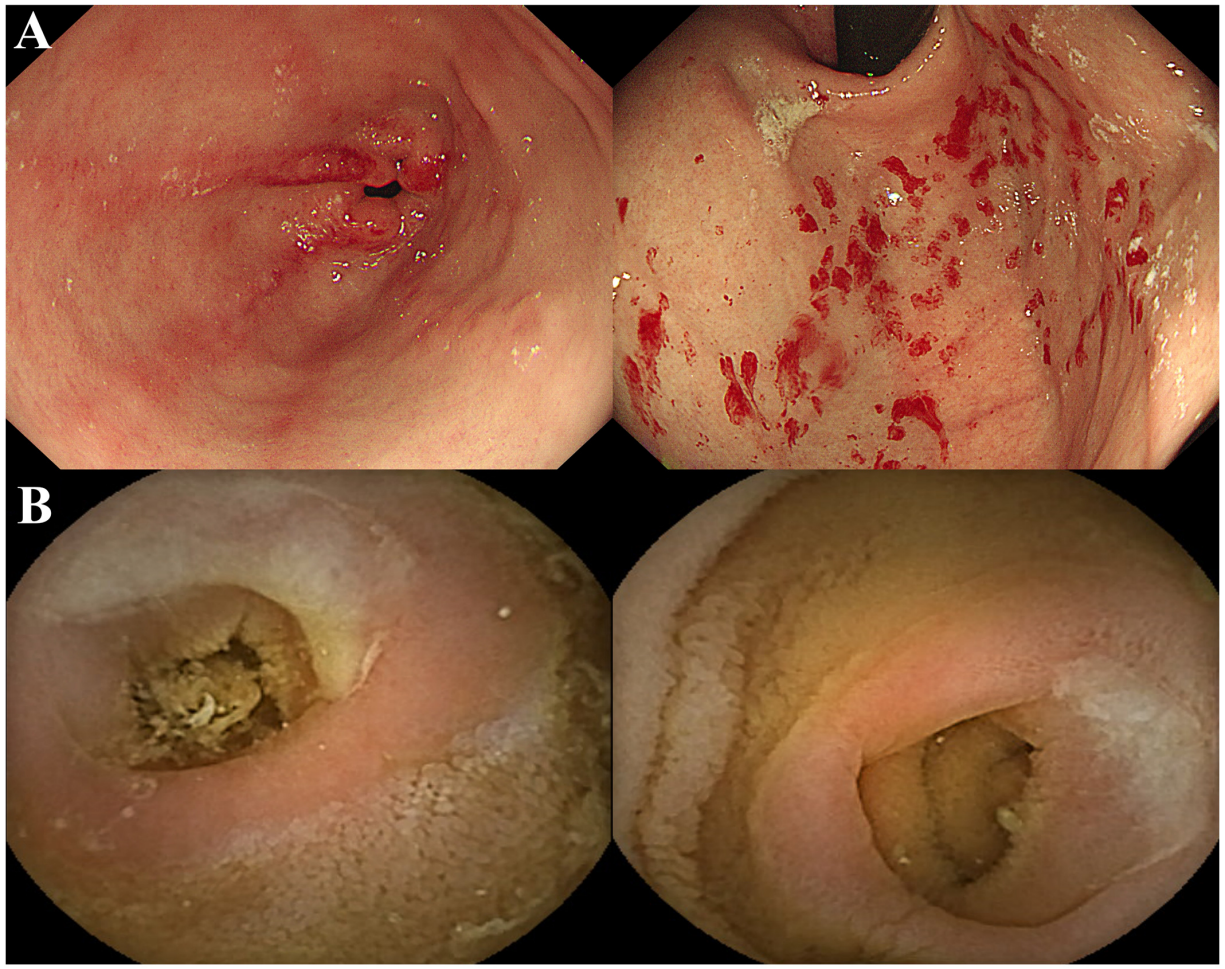
**(A)** Upper endoscopy showing erythematous changes in stomach antrum and body. With other findings unremarkable, the impression was erythematous gastritis. **(B)** Following capsule endoscopy showed chronic inflammation, multiple ulcers and strictures in small bowel. No bleeding focus was found.

Two months later, a follow-up endoscopy revealed chronic erythematous gastritis without significant eosinophil infiltration or H. pylori infection (Figure 1A). Given the possibility of allergic eosinophilic gastroenteritis related to food, a MAST test was conducted, and a diet restricting tomatoes and eggs (which tested positive) along with antihistamines was tried. However, stool calprotectin and α1-antitrypsin levels remained elevated, indicating limited efficacy.

Ultimately, a small bowel capsule endoscopy was performed, and the results revealed chronic inflammation, ulcers, and multifocal strictures in several areas of the small intestine (Figure 1B). The capsule remained in the small intestine for over 12 h, unable to pass through the narrowed sections. These findings raised suspicions of Crohn's disease, Cryptogenic multifocal ulcerous stenosing enteritis (CMUSE), or Chronic nonspecific multiple ulcers of the small intestine (CNSU). In the meantime, while the genetic testing for short stature was being conducted, a heterozygous variant classified as a Variant of Unknown Significance (VUS) in the SLCO2A1 gene (c.95A>G) was reported. In a family genetic test, the mother tested positive for the mutation in the same gene and the father tested negative. And another SLCO2A1 gene heterogenous c.1149del mutation was detected and classified as a likely pathogenic mutation. Results of the parents' genetic testing about SLCO2A1 gene c.1149del mutation are pending.

Correlating this result with the clinical findings ultimately led to the diagnosis of chronic nonspecific multiple ulcers of the small intestine (CNSU), which has been renamed as chronic enteropathy associated with the SLCO2A1 gene (CEAS). Serial x-ray was taken after the capsule endoscopy, but the capsule was retained in the same location even after 19 days. Since it did not seem likely to pass through the narrowed area, we eventually decided to proceed with surgical removal.

**Figure 2:**
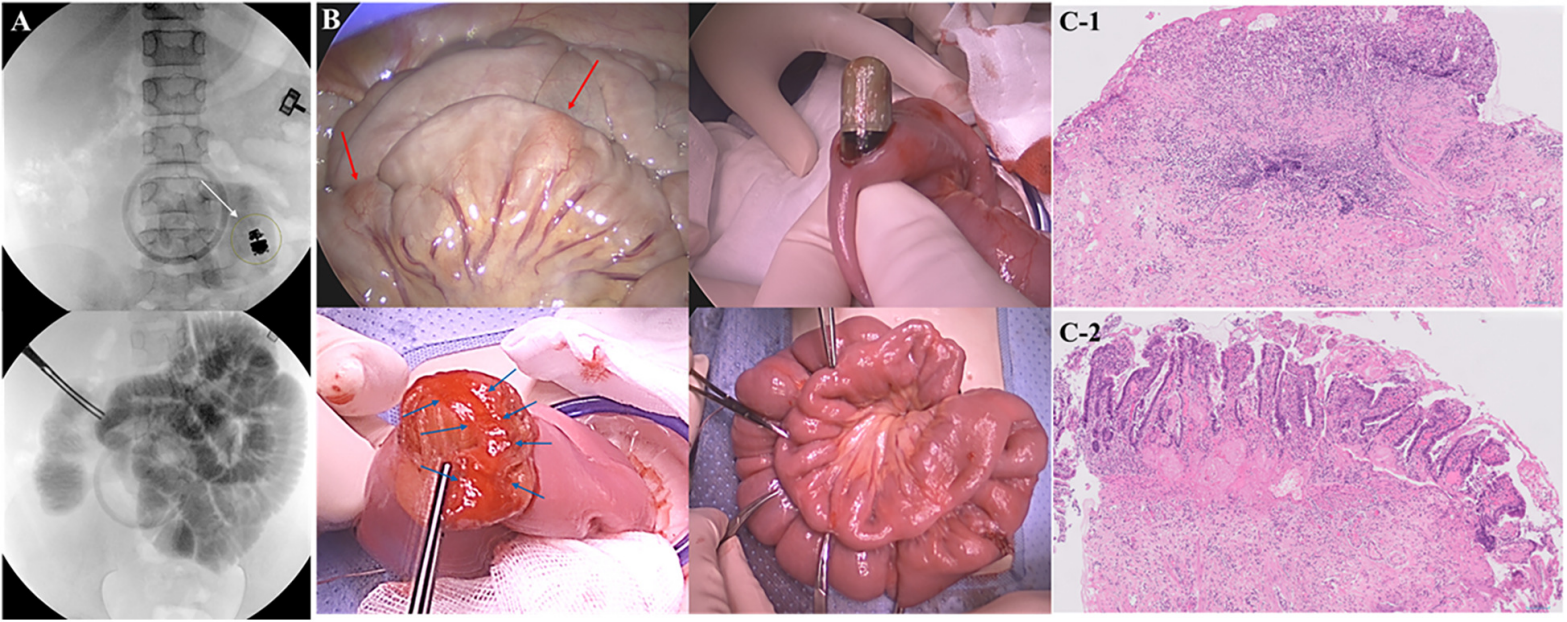
Intraoperative images during 1st surgery. **(A)** C-arm was used to locate the capsule, which was found 2 m above the IC valve (white arrow). Iobrix was injected to further confirm the proper bowel passage. **(B)** After retrieving the endoscope, enteroplasty was done. Small bowel was narrowed in a ring-like manner (red arrow), and along the incision site, band-like ulcers (blue arrow) were observed. **(C)** Representative histologic findings of the ileum. **(C-1)** A shallow ulcer with inflammatory infiltrate, confined to the mucosa and submucosa. **(C-2)** An adjacent erosive area exhibiting mucosal denudation and marked vascular congestion. (H&E stain, original magnification ×10).

During the surgery, a C-arm fluoroscope was used to locate the capsule endoscope, which was found 2 m above the ileocecal valve. A 3 cm incision was made at the stricture site to retrieve the endoscope, and enteroplasty was done. Extensive band-like ulcers, spaced 2 cm apart, were observed along the incision site (Figure 2). A partial resection of the small bowel mucosa was performed along with biopsy, and after confirming proper passage with iobrix injection, the surgery was completed. The final pathology was revealed as chronic active inflammation with ulceration, abscess formation, and fibrosis.

**Figure 3:**
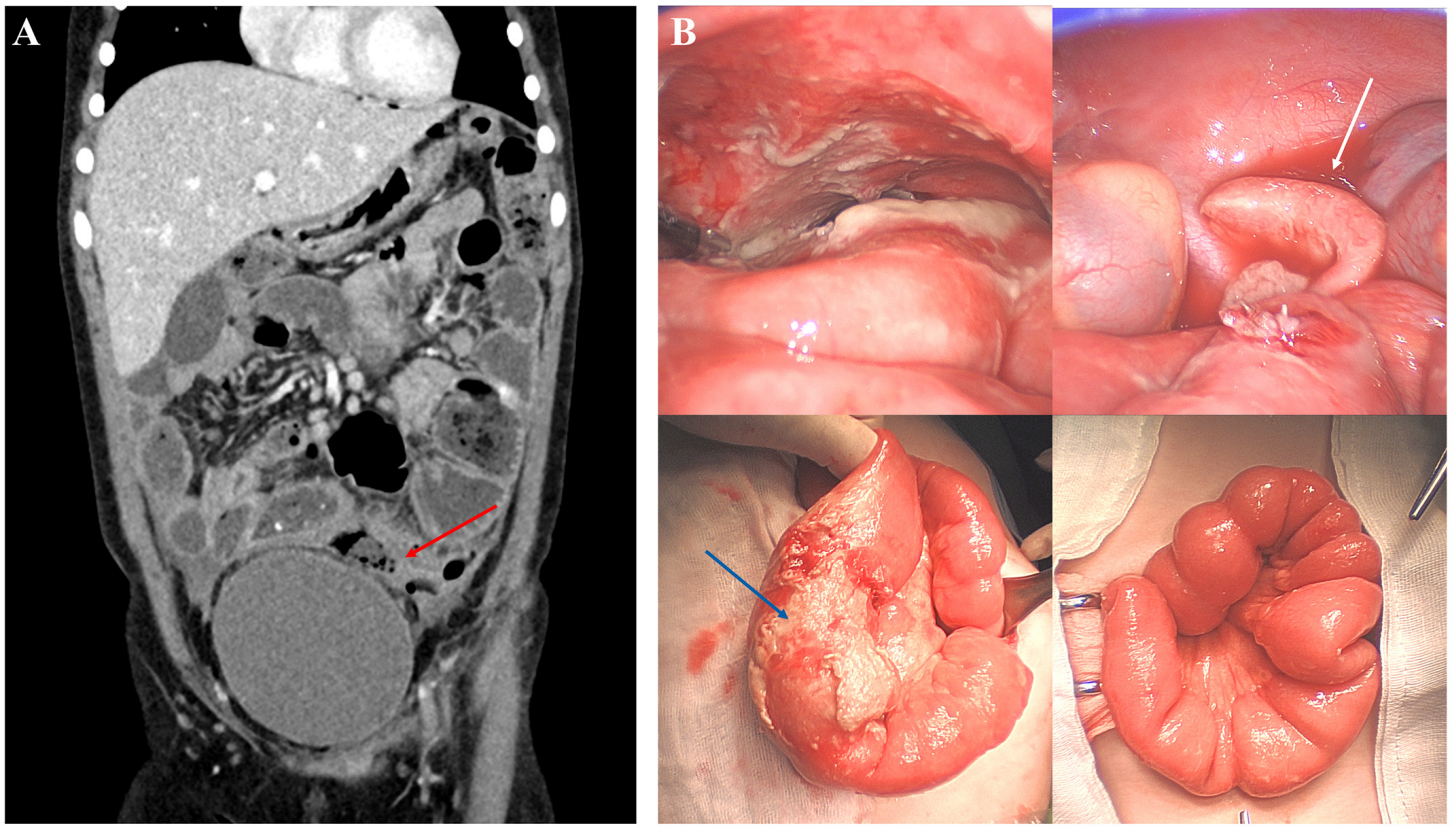
Imagings before and during 2nd surgery. **(A)** CT scan showing intraperitoneal free air and multifocal abscess in pelvic cavity, suggesting bowel perforation. Possible perforation site was sigmoid colon or jejunoileal junction (red arrow), but not definite. **(B)** Intraoperative images of pelvic inflammation and abscess. Appendix was swollen (white arrow), and suspected sealed-off perforation site was reinforced (blue arrow). Ring-like strictures were still noted as before.

After discharge, the patient began steroid and immunosuppressive therapy (Azathioprine) in accordance with the diagnosis of CEAS. Steroid was tapered after one month, and the patient had been maintaining on Azathioprine alone. Three months after starting the immunosuppressive therapy, the patient visited the emergency room with high fever and abdominal pain. The abdominal pain had begun three days prior and was worsening, with fever and vomiting starting the previous day. Upon admission, her body temperature was measured 38.6°C and the physical examination revealed generalized lower abdominal tenderness and rebound tenderness. Initial laboratory findings showed a WBC count of 12,800 × 10^6^/L, CRP of 37.3 mg/dl, and procalcitonin of 19.98 ng/ml. The initial abdominal CT scan revealed free air throughout the abdomen and multifocal abscess cavities, with suspicious perforation site at jejunoileal junction or sigmoid colon, although definite identification was unclear (Figure 3A). Emergency surgery was performed.

An abscess cavity was located just below the umbilicus with severe pelvic adhesions due to pus and inflammatory fibrotic tissue. No definitive perforation site was identified upon tracing the whole bowel, including the previous enteroplasty site, suggesting it was sealed off. The small bowel was narrowed in a ring-like manner, measuring approximately 110 cm from 120 cm below the ligament of Treitz, with no issues regarding bowel passage, so strictureplasty was not performed (Figure 3B). The suspected perforation site was in the distal jejunum, adhered to sigmoid colon. After adhesiolysis and reinforcement sutures, the swollen appendix, likely due to secondary changes, was resected. The procedure concluded with extensive irrigation.

**Figure 4:**
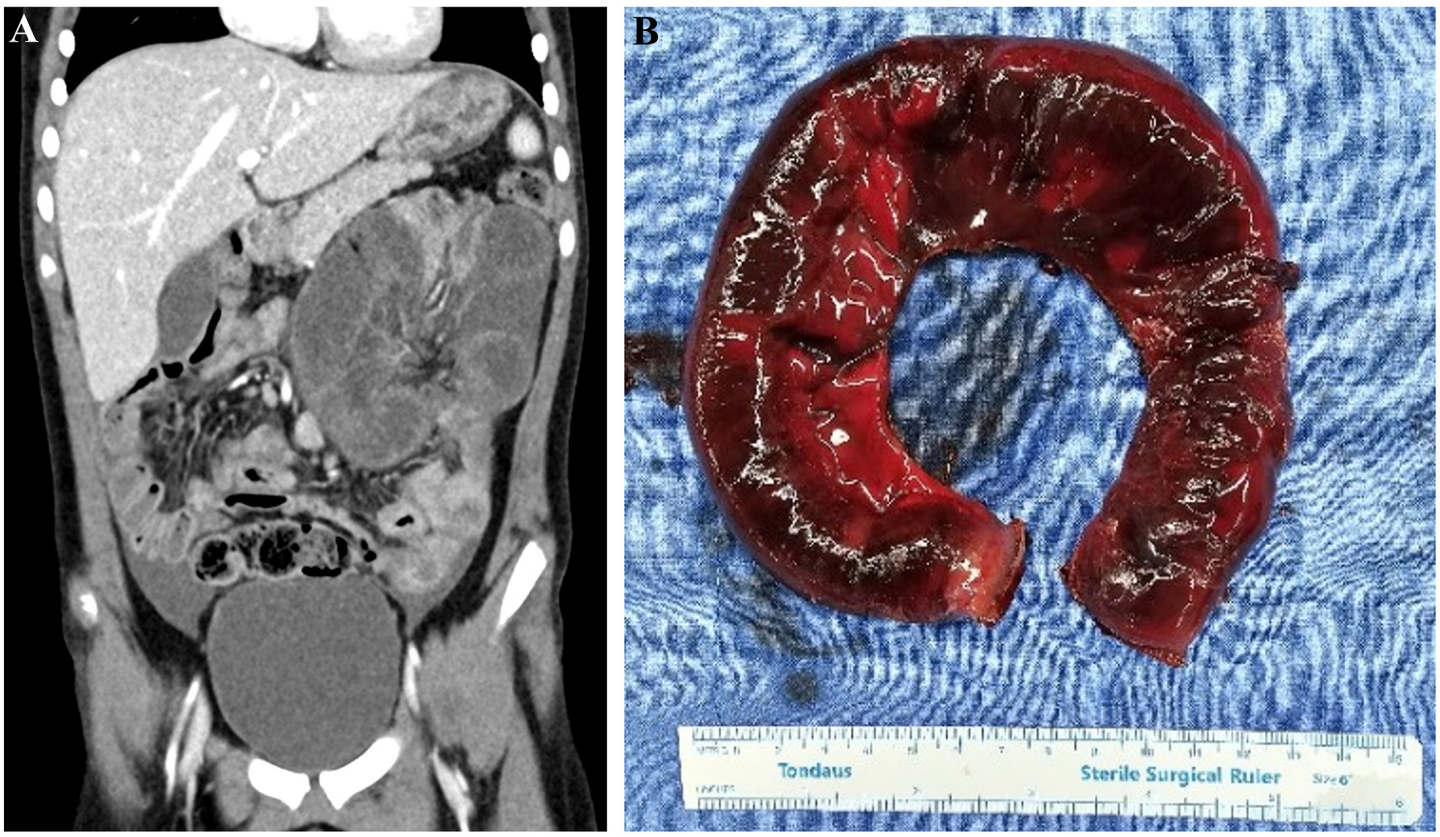
Imagings before and during 3rd surgery. **(A)** CT scan showing marked distension of small bowel loops and decreased enhancement, indicating closed loop obstruction with ischemia. **(B)** Intraoperative image of resected jejunum. Apart from ischemia, the diffuse circular stenoses found in the previous operations seemed almost disappeared.

The patient was discharged after about 10 days of intravenous antibiotics, and after two weeks, a step-up treatment with infliximab was initiated. Infliximab was given in two-week intervals without complications. Then, few days after the second infusion, she visited the emergency room with sudden projectile vomiting. There was no abdominal pain or fever, but the initial lab results showed an elevated WBC count of 17,420 × 10^6^/L and lactic acid of 5.1 mmol/L. A CT scan was performed, presenting suspicion of ischemia due to closed-loop obstruction (Figure 4A), so urgent surgery was conducted. During the operation, multiple fibrotic bands were observed causing approximately 40 cm of the small intestine to be twisted, 60 cm below the Treitz ligament. After warm saline irrigation, partial viability was restored, and 35 cm of the ischemic portion of jejunum was resected and anastomosed (Figure 4B). A significant amount of fibrotic adhesions were noted, but the overall condition of the bowel, including the circular stenoses, showed much improvement compared to the earlier findings, and bowel passage was unimpeded. The final pathology review also confirmed that, compared to the severe ulceration observed in the mucosa of the initial surgery, there was almost no ulceration in the specimen from this surgery. The conclusion was that the last surgery was not due to the worsening of the disease; In fact, the bowel condition seemed to be actually improving due to infliximab. Rather, the post-operative adhesion was the cause, triggered by overeating as a leading factor for obstruction. After starting liquid diet on the second post-operative day, and with fine laboratory results, the patient was discharged in a tolerable state one week after surgery. Based on the findings from the last surgery, she is planning to keep on infliximab treatment primarily, and is scheduled for outpatient follow-up.

## Discussion

Apart from traditional diseases affecting the small intestine such as Crohn's disease, intestinal tuberculosis, eosinophilic gastroenteritis or NSAID enteropathy, research on idiopathic superficial ulceration and stenosis of the small intestine has begun in the last few decades (1). These conditions characteristically affect only the small intestine, forming nonspecific ulcers and causing chronic blood and protein loss. Such distinct ulcerative diseases are referred to as chronic nonspecific multiple ulcers of the small intestine (CNSU) or cryptogenic multifocal ulcerous stenosing enteritis (CMUSE). In 2015, Umeno et al. first identified an inherited pattern of CNSU caused by a loss of function mutation in the SLCO2A1 gene, which was subsequently termed chronic enteropathy associated with the SLCO2A1 gene (CEAS) (2).

The pathophysiology is not yet fully understood; however, SLCO2A1 is a gene that encodes a prostaglandin transporter, and prostaglandins are known to be involved in regulating inflammatory responses in the gastrointestinal tract and in mucosal healing (3). Additionally, one study found that loss of function of SLCO2A1 increases PGE2 levels, which in turn enhances macrophage activity. Other studies have revealed that SLCO2A1 protein is not observed in the vascular endothelial membrane of the CEAS patients' small intestine. Through this overall mechanism, it is hypothesized that the gastrointestinal tract barrier becomes compromised; however, further research is still needed.

CEAS occurs predominantly in females and mostly in adolescents, but also known to be found in people aged 1–69 years (4, 5). The main clinical symptoms include general fatigue, edema, and abdominal pain. Key laboratory features suggest iron deficiency anemia (IDA), hypoalbuminemia, and near-normal levels of CRP (6). Endoscopic findings reveal multiple ulcers or strictures confined solely to the small intestine, with the most commonly affected area being the ileum, while sparing the terminal ileum. Unlike the symmetric mural abnormality with mesenteric dominance seen in Crohn's disease, the characteristic findings of the ulcers typically show clean margins and an asymmetrical pattern (7, 8). Additionally, because the ulcers primarily occur superficially in the mucosa and submucosal areas, there is no marked elevation in systemic inflammatory markers such as ESR or CRP, and microscopic occult blood tests may be positive without gross hematochezia (9).

Although there are some distinguishing features, not all patients present in a typical way and since CEAS itself is not common, it is difficult to make an early diagnosis of CEAS. Most patients are often approached in the manner of Crohn's disease, and when medical treatment turns out to have no response, then other idiopathic diseases like CEAS are suspected. The patient in our case also followed this usual pattern. Initially, she underwent workup for IDA and hypoalbuminemia, and with suspicion of other enteropathies, she attempted treatments like antihistamine and dietary modifications but none showed any effect. During genetic testing conducted to investigate the cause of her growth retardation, a mutation in SLCO2A1 gene was identified. Correlating this finding with the multiple ulcers and strictures observed in capsule endoscopy, a final diagnosis of CEAS could be established. Despite these difficult approaches, it is crucial to consider CEAS in the differential diagnosis when faced with atypical clinical findings, particularly in patients presenting with unexplained gastrointestinal symptoms. In these scenarios, genetic testing emerges as a valuable diagnostic tool, allowing for a more accurate identification of CEAS and facilitating timely and appropriate management for affected patients.

In addition, a clear treatment for CEAS remains elusive. Usually, when corticosteroids are ineffective or there is a relapse, it is common to try immunomodulators like 5-aminosalicylic acid (5-ASA) and azathioprine (10). There are reports of remission with biologics like anti-TNF treatment, but the data is limited (11–13). Long-term follow-up results indicate that most patients end up requiring surgery, and due to frequent recurrence, many cases report undergoing multiple surgeries (14). The patient in our case underwent three surgeries while maintaining medical treatments; each for stricture, bowel perforation leading to panperitonitis, and obstruction due to adhesion band. The inevitable long term immunosuppressive therapies can result in complications requiring surgery, such as fistula, abdominal abscess, perforation, stenosis, or obstruction (15). Therefore, considering the possibility of multiple surgeries and to prevent short bowel syndrome, the surgeon should always consider a minimally invasive approach for the patient.

Additionally, the resolution of circular stenosis seen in the final surgery, performed while the patient was receiving infliximab treatment, suggests that there may be a reversible component. To our knowledge, there has been no documented report of such visual confirmation regarding effective treatment for CEAS, making the surgical findings in this case clinically significant. Additionally, the possibility that the stricture itself may represent a reversible finding draws attention to its pathophysiology. For instance, previous studies have indicated that infliximab primarily targets the mucosal level, promoting the mucosal healing (16). In contrast to Crohn's disease, which involves the smooth muscle layer and leads to chronic inflammation, CEAS is characterized by ulcers only confined to mucosa and submucosa. This distinction could possibly explain the differences in drug response observed in this case. Of course, further follow-up is necessary to confirm this observation. However, we believe this finding has suggested possibilities for CEAS patients who are refractory to both steroid and immunomodulators.

Since CEAS is a disease that has only recently been identified, there are limited research especially in pediatric cases. In South Korea, the first patient was reported in 2018, followed by the first case series study involving 14 patients published in 2020 (17). There is only one confirmed report involving pediatric patients in South Korea, and no reported case of CEAS patient undergoing as many as three surgical interventions, as seen in our case (6). Prior studies on CEAS have primarily focused on describing the distinctive disease entity and diagnostic approaches, and little is discussed about the treatment and prognosis, particularly from a surgical perspective (18). Therefore, I believe our case holds significance, and further study will be necessary based on treatment response and long-term prognosis.

## Data Availability

The original contributions presented in the study are included in the article/Supplementary Material, further inquiries can be directed to the corresponding authors.
